# Pellet Production from Pruning and Alternative Forest Biomass: A Review of the Most Recent Research Findings

**DOI:** 10.3390/ma16134689

**Published:** 2023-06-29

**Authors:** Rodolfo Picchio, Nicolò Di Marzio, Luca Cozzolino, Rachele Venanzi, Walter Stefanoni, Leonardo Bianchini, Luigi Pari, Francesco Latterini

**Affiliations:** 1 Department of Agriculture and Forest Sciences, DAFNE, Tuscia University, Via San Camillo de Lellis, 01100 Viterbo, Italy; nicol.dimarzio@studenti.unitus.it (N.D.M.); venanzi@unitus.it (R.V.); l.bianchini@unitus.it (L.B.); 2 Consiglio per la Ricerca in Agricoltura e l’Analisi dell’Economia Agraria (CREA), Centro di Ricerca, Ingegneria e Trasformazioni Agroalimentari, Via della Pascolare 16, 00015 Monterotondo, Italy; luca.cozzolino@crea.gov.it (L.C.); luigi.pari@crea.gov.it (L.P.); 3 Research Institute on Terrestrial Ecosystems (IRET), National Research Council (CNR), Via Salaria Km 29.300, 00015 Monterotondo, Italy; walter.stefanoni@cnr.it; 4 Institute of Dendrology, Polish Academy of Sciences, Parkowa 5, 62-035 Kórnik, Poland; latterini@man.poznan.pl

**Keywords:** bark, lignocellulosic biomass, bioenergy, renewable energy, short-rotation coppice

## Abstract

Typically, coniferous sawdust from debarked stems is used to make pellets. Given the high lignin content, which ensures strong binding and high calorific values, this feedstock provides the best quality available. However, finding alternative feedstocks for pellet production is crucial if small-scale pellet production is to be developed and used to support the economy and energy independence of rural communities. These communities have to be able to create pellets devoid of additives and without biomass pre-processing so that the feedstock price remains low. The features of pellets made from other sources of forest biomass, such as different types of waste, broadleaf species, and pruning biomass, have attracted some attention in this context. This review sought to provide an overview of the most recent (2019–2023) knowledge on the subject and to bring into consideration potential feedstocks for the growth of small-scale pellet production. Findings from the literature show that poor bulk density and mechanical durability are the most frequent issues when making pellets from different feedstocks. All of the tested alternative biomass typologies have these shortcomings, which are also a result of the use of low-performance pelletizers in small-scale production, preventing the achievement of adequate mechanical qualities. Pellets made from pruning biomass, coniferous residues, and wood from short-rotation coppice plants all have significant flaws in terms of ash content and, in some cases, nitrogen, sulfur, and chlorine content as well. All things considered, research suggests that broadleaf wood from beech and oak trees, collected through routine forest management activities, makes the best feasible feedstock for small-scale pellet production. Despite having poor mechanical qualities, these feedstocks can provide pellets with a low ash level. High ash content is a significant disadvantage when considering pellet manufacture and use on a small scale since it can significantly raise maintenance costs, compromising the supply chain’s ability to operate cost-effectively. Pellets with low bulk density and low mechanical durability can be successfully used in a small-scale supply chain with the advantages of reducing travel distance from the production site and storage time.

## 1. Introduction

There is a lot of interest in renewable energy sources as a result of the steady decline in the availability of fossil fuels as energy sources and the severe environmental issues associated with their use, as well as the current geopolitical situation [1,2]. Biomass is unquestionably one of these sources [3,4].

The most common sources of biomass for bioenergy include forest biomass (both residues and low-value wood) and biomass from orchard pruning. Biomass from forest interventions suitable for bioenergy production is generally named fuelwood [5]. Fuelwood is gathered from forested areas and either burned directly to produce heat or transformed into bioenergy and biofuel to produce heat and then power. More specifically, fuelwood is a viable feedstock for thermochemical conversion, biological conversion, liquefaction, and gasification due to the high amounts of macromolecular carbohydrates, like cellulose and organic matter [6]. In boilers and other power-generating machinery, forest biomass can be used either alone or in conjunction with fossil fuels [7,8].

More than 10 million hectares are covered with fruit orchards in the European Union alone, the majority of which are found in Mediterranean regions [9]. Every orchard needs routine pruning, which is undertaken every one to three years. This operation produces significant amounts of residues, estimated to be between 1 and 5 tons per hectare [10]; over 2600 kt dry matter of pruning biomass is produced annually just from Italian olive groves [11]. This is a potentially significant source of biomass for energy production [12]. However, these leftovers are typically mulched or burned in the field [13,14]. No economic advantage can be obtained with this method [15]. Additionally, field burning results in greenhouse gas (GHG) emissions. Mulching has benefits such as lower soil erosion and soil nutrient depletion, but there is also the negative aspect of a higher risk of disease transmission [16,17]. Therefore a marked interest has come about in using biomass from orchard pruning to produce bioenergy [18,19].

**Figure 1:**
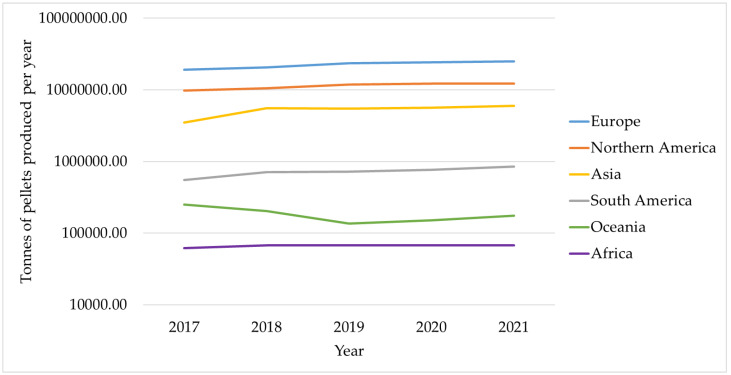
Production of pellets in the various continents from 2017 to 2021 [32]. The *y*-axis is logarithmic to account for the high variability in production in the various continents.

The main issues with using any kind of biomass for energy production are related to its low bulk density and high moisture content, which make handling, transporting, and storage challenging [20,21,22,23]. Densification and standardization of solid biofuels, which are likewise characterized by higher energy density, are current trends in addressing the critical problems with the use of biomass for energy [24,25]. Although there are currently many densification methods available, pelletization is the one that is most frequently applied [26]. Using biomass in the form of pellets results in biofuel that is more affordable than when using biomass waste directly for energy generation. Pellet production takes place by extrusion, which involves passing semi-dry biomass that has been previously processed into dust, sawdust, or shavings through a hole that is a few millimeters in size to create small cylinders that are cut to the necessary length and then cooled [27,28]. This procedure lowers the cost of handling, transport, and storage by increasing the bulk density of the biomass [29,30,31]. The statistics from FAOSTAT [32], which illustrate the rising level of pellet production globally (Figure 1), highlight the significant importance of the pelletization process for energy production. Analyzing a biofuel’s quality is one of the key components of using it correctly and sustainably. The characteristics of the raw material utilized, such as its chemical composition, moisture content, and particle size distribution, as well as the operating circumstances, such as the die temperature, applied pressure, and holding duration, all have an impact on the quality of the pellets [33,34]. Additionally, pellets can be made from a variety of feedstocks, mostly those connected to forestry and agricultural activities [35,36]. It is much more crucial to evaluate the quality of pellets by taking into account the wide range of raw materials that can be used for pelletizing [37].

Among all the different types of biomass, wood serves as the primary feedstock for the manufacturing of pellets [38]. Sawdust is a perfect substrate since it is unprocessed and even small pollutants can be eliminated by removing the bark and cleaning the logs before sawing [39]. However, there has been considerable interest in and investigation into the creation of pellets with different techniques and sources due to the rising demand for wood pellets and the restricted supply of sawmill residues [40].

**Figure 2:**
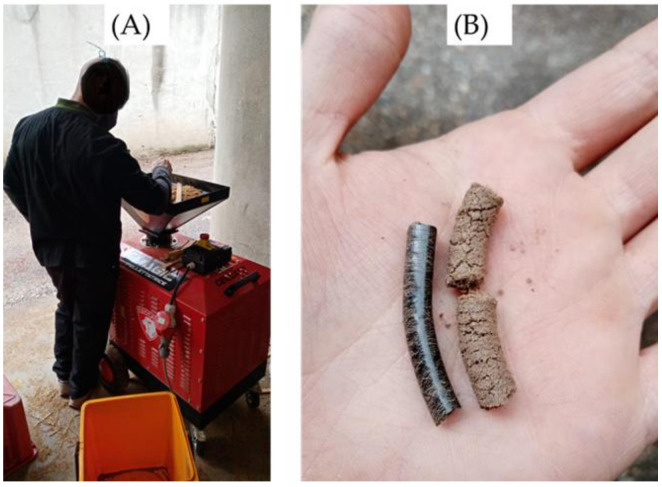
(**A**) Small-scale pellet production. (**B**) High- (on the left) and low-quality (on the right) pellets.

As with any biomass supply chain, pellet production can be implemented at the industrial level or on a small scale. The implementation of industrial production of pellets requires high amounts of available feedstocks located in a concentrated area to limit transport costs and environmental concerns [41,42]. On the other hand, recent studies have highlighted the importance of implementing small-scale production of pellets based on local material deriving from single or limited groups of producers [43]. Small-scale pellet production in rural areas can also help local farmers and small- to medium-sized forestry businesses by increasing their income [44,45,46]. To accomplish this, it is crucial to assess the quality of the pellets made from the raw material as it is (Figure 2). On an industrial scale, high-value pellets are often made from pure sawdust [47], and some further processing steps, such as pre-treatment and the addition of binders, are also applied [26]. Comparatively, pellets produced from various feedstocks that contain significant amounts of bark or leaves may be of lower quality [48,49]. In the small-scale production of pellets carried out by small farmers, forest/sawmill owners or forest enterprises must use time, energy, and money to pre-treat the biomass, and the pellet production should take place with raw feedstocks that are located with the least distance possible from the biomass harvesting site [43,50].

This review seeks to provide the reader with a comprehensive overview of the most recent knowledge on this topic. We focused on papers published from 2019 to 2023 that studied the quality of pellets produced from pruning and alternative forest biomass (bark, branches, short-rotation coppice residues, and alternative species) without considering any additives or pre-treatment of the biomass other than some degree of mixture with coniferous sawdust. The influence of the biomass properties on pellet quality was the main topic of the current review, which must be emphasized. The impacts of the process variables (die temperature, applied pressure, and holding time) on the characteristics of the pellets were not included in this paper because they are a separate, very broad topic that requires a separate analysis.

The review is structured as follows: after the present introduction, we provide a description of the literature search criteria and implementation. Secondly, we report the quality standards for pellet evaluation. Then, we treat the papers dealing with pellet production from forest biomass and from pruning separately, and we conclude with a dedicated section in which we try to make some suggestions for future research.

## 2. Materials and Methods

The first step to identify literature sources was a search within the Web of Science and Scopus databases to retrieve the most recent publications on the topic. We used as keywords: “pellet*”; “forest biomass*”; “forest residue*”; “pruning*”; “densification”; “short rotation forestry”; and “short rotation coppice”. We used the Boolean operators “AND” and “OR” to link the keywords. In detail, the applied search keys were: “pellet*” AND “forest biomass*”; “pellet*” AND “forest residue*”; “pellet*” AND “pruning*”; “pellet*” AND “short rotation forestry”; “pellet*” AND “short rotation coppice”; “densification” AND “forest biomass*”; “densification” AND “forest residue*”; “densification” AND “pruning*”; “densification” AND “short rotation forestry”; and “densification” AND “short rotation coppice”.

We referred only to papers in English published from 2019 to April 2023. We obtained further literature references by using the snowball approach [51,52], thus relying on the reference lists of the most recently published papers. In particular, we applied the snowball method to the literature references from an initial amount of five papers. The method was applied in just one step, as no further manuscripts were detected after the first step.

We focused on papers that reported the production of pellets without treatments such as torrefaction and without using additives. Moreover, we included in the database for review only papers that analyzed pellet quality according to the standard EN ISO 17225-2 and not those focusing, for instance, only on emission analysis. In this way, we built a database for review made up of 29 papers, 10 of which focused on pellets from pruning and 19 on pellets from alternative forest biomass.

## 3. The Standards for Pellet Quality Assessment

The International Organization for Standardization (ISO) has created global pellet quality standards. To name a few, EN ISO 17225-1 covers general quality requirements, EN ISO 17225-2 covers graded wood pellets for residential and commercial usage, and EN ISO 17225-6 covers graded non-woody pellets. The Austrian standard NORM M 7135, the Swedish standard SS 187120, the German standards DIN 51731 and DIN EN 15270, the Italian standard CTIR04/05, and the French recommendation ITEBE represent just a few of the European nations that have previously developed laws and standards for pellet quality certification [53]. The EN ISO 17225 set of ISO fuel specification standards, which took the place of EN 14961, was released in May 2014.

The usage of pellets for both industrial and non-industrial purposes is covered by the graded wood pellet standard (EN ISO 17225-2). Use of fuels in smaller appliances, such as those found in homes, small commercial establishments, and government structures, is referred to as non-industrial use [54]. The best quality class according to this guideline is A1, which refers to virgin wood and chemically undisturbed wood residue low in ash and nitrogen. Pellets classified as A2 have slightly higher nitrogen and ash contents. Property class B comes last. Chemically processed industrial wood byproducts and residue fall under this category [54]. A classification of pellets for industrial usage is also reported in ISO 17225-2. Three alternative quality classes (I1, I2, and I3) are provided by this classification, and they have significantly stricter requirements than classes A1, A2, and B for pellets intended for household use. The introduction of this standard at the European level marked a significant development for the industry by guaranteeing better product transparency throughout products’ entire evolution and enabling greater conformity with global markets.

Standards for non-woody pellets (ISO 17225-6) cover pellets formed from mixtures and blends, such as biomass from herbaceous plants, fruits, or aquatic life. Two classification tables are provided by this standard: one for pellets made of straw, miscanthus, and reed canary grass and the other for biomass and blends of herbaceous and fruit materials. Non-woody pellets typically have higher levels of ash, chlorine, nitrogen, and sulfur [54], as well as a lower heating values (LHVs). Since the required standard is less stringent and they can have lower quality levels than wood pellets, it would be preferable to pay more attention to clearly communicating qualitative differences and usage suggestions. Given the high level of dynamism in this industry, it is critical that robust biofuel standards continue to be developed.

A summary of the requirements of ISO 17225-2 is given in Table 1.

## 4. Pellets from Alternative Forest Biomass

Basically, pellets are mostly already produced from forest residues; in particular, with coniferous sawdust derived from sawmills. However, there has been great interest in recent years in producing pellets from alternative species, both from natural stands and from dedicated plantations, and from other types of forest residues, such as bark, cones, or material from low-diameter wood like branches [55,56].

In a recent study examining the production of pellets by adding cones and bark to spruce sawdust, it was found that the addition of these residues lowered the overall quality of the produced pellets, mostly in terms of ash melting behavior, nitrogen content, and ash content [57]. However, the major part of the produced mixtures reached the quality standards for at least the domestic B class, apart from ash melting behavior, for which the melting point was too low [57]. Terzopoulou et al. [58] confirmed that a low percentage (<7%) of cypress bark should be kept in feedstock for pellet production to achieve satisfactory quality. In another trial that investigated the possibilities of using stone pine (*Pinus pinea* L.) bark, medium branches, thin branches, and needles to produce pellets, the authors revealed that it was not possible to achieve high enough quality by only using these alternative feedstocks [59]. Higher quality could be achieved by mixing them with stone pine debarked wood in a certain ratio that varied based on the type of residue (about 15% for bark, 30% for medium branches, and less than 15% for needles and thin branches), which could yield the highest-quality pellets [59]. The authors recommended using the thick wood (trunk plus thick branches), as well as a portion of the medium branches and bark. It would be more practical to leave the needles and thinnest branches in the forest for their incorporation into the soil due to their high nutrient concentration and poor quality for energetic uses [59].

Focusing on pellet production from broadleaf species, *Quercus* spp. pellets produced in various trials across the world with different species reached generally satisfactory results. Carrillo-Parra et al. [60] produced pellets from three Mexican oak species and achieved satisfactory heating values and low ash content, as well as good mechanical durability, which made it possible to achieve the quality standards for domestic use. Pellets from the same Mexican oak species could be further improved by adding up to 20% coniferous sawdust from debarked stems [61]. Regarding European oak species, pellets produced from residues from urban green area management of *Quercus ilex* L. reached quality standard A2 for the heating value and also the minimal requirement for bulk density [62]. *Quercus robur* L. pellets showed satisfactory heating values and ash content when pelletizing feedstock with low initial moisture content [63]. In contrast, in another study, *Quercus robur* L. did not achieve the quality standard for the heating value, bulk density, and mechanical durability as a consequence of low lignin content when compared to coniferous oak wood [64]. Results for beech pellets are very similar to those reported for oak ones, and different studies highlighted satisfactory results [50,63,65], even if Stolarski et al. highlighted excessively low heating values, bulk density, and mechanical durability [64]. Pellets produced from poplar and birch showed generally high ash content, making them suitable only for industrial applications [64,66]. Similar results were reported for eucalyptus bark pellets, which showed a high level of fines [67].

**Figure 3:**
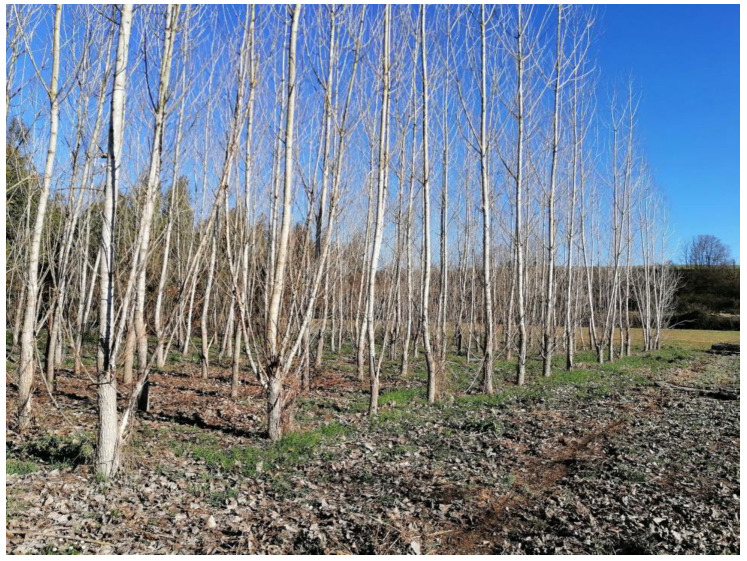
Poplar medium-rotation coppice plantation. Although ensuring lower bark/wood ratio and subsequent lower ash content than short-rotation coppicing, the dimensions of the stems require different harvesting systems.

Concerning pellet production from short-rotation coppice plantations, the literature shows that pellets obtained from this form of management are generally suitable only for industrial uses; they reach only B class for domestic applications [65,68]. The main problem is related to the low diameter of the shoots, which results in a high bark/wood ratio [63]. Increasing the rotation cycle to medium-rotation coppicing (MRC; about six to seven years for rotation; Figure 3) generally leads to an increase in the quality of the pellets obtained. This has been confirmed for both poplar and eucalyptus MRC plantations compared to SRC ones [50,69]. However it is worth highlighting that increasing the rotation time leads to higher dimensions for the stems to be harvested, thus no longer allowing for single-passage harvesting but requiring double-passage harvesting carried out with machinery specifically developed for forest management (chainsaw, feller-buncher, harvester, forwarder, cable skidder) [70,71,72,73]. Apart from low bulk density and mechanical durability, some authors have also found excessive nitrogen, sulfur, and chlorine as a consequence of the fertilization activity carried out in SRC plants [74]; however, this strongly depends on the plantation management adopted and on the specific characteristics of the growing site.

Finally, it is worth highlighting that, in view of small-scale pellet production, low mechanical durability and bulk density can also be related to the usage of low-cost, small-scale pelletizers that do not have the capacity to reach the compressive force and working performance of industrial machinery for pellet production [75].

In summary, it is obvious that the best-quality pellets are produced from pure coniferous sawdust derived from debarked stems. However, different types of forest biomass can be used for pellet production, even if there are some technical limitations. The characteristics that are mainly affected when producing pellets from alternative feedstocks are mechanical durability and bulk density, considering that bark, cones, leaves, needles, and broadleaf wood all have lower lignin content than coniferous sawdust. Lignin is the main factor in the binding process in pellet production [67]; therefore, it is understandable that materials with lower lignin content produce pellets with worse mechanical properties. Sawdust from broadleaf species, particularly oak species, generally contains 13–16% lignin, while in pine sawdust, the share of lignin can reach 26.3% [60].

Lignin also has a high heating value [76,77], and this also explains why some of the pellets produced from alternative feedstocks show lower heating values as compared to coniferous pellets. This is also explainable considering that coniferous wood also contains resin, which can increase the heating value [63]. However, low bulk density and mechanical durability may not be an insurmountable obstacle in developing a small-scale pellet supply chain. Bulk density is a parameter related to transport cost, and keeping a short distance between the pellet production site and the plant, approximately within a radius of 10 km [22], can limit the impact of the low bulk density of the produced pellets. A pellet with low mechanical durability may be less resistant to abrasion stress during handling, transport, and storage because of the mechanical load but may also show higher moisture absorption [78]. This could increase the risk of fire and explosions [79]. However, limiting transport distance and storage time could considerably decrease this risk. On the other hand, pellets with high ash content can represent a major problem from the perspective of small-scale production and utilization, leading to a substantial increase in maintenance costs [80]. Considering the above, wooden material from oak and beech trees seems to be a more interesting feedstock for small-scale pellet production than coniferous residues and SRC biomass, given that the first show low mechanical properties but low ash content while the latter has high ash content and low mechanical properties.

## 5. Pellets from Pruning

Pruning is even more challenging than alternative forest biomass for the production of pellets. In fact, pruning residues are characterized by low diameter, a high bark/wood ratio, and the presence of leaves and sometimes material derived from wood attacked by pathogens, as well as material contaminated with soils, as a consequence of pruning mechanical harvesting [81]. Furthermore, the possibility that pruning biomass could contain high levels of heavy metals as a consequence of fertilization and chemical management of pests can also raise some important environmental concerns [82]. Notwithstanding these challenges, there is wide interest in the valorization of pruning biomass through pelletization considering that the low bulk density of pruning biomass is the major challenge for the implementation of effective biomass supply chains [83,84].

In the database for this review, the major part of the studies dealing with the production of pellets from pruning biomass were conducted in the Mediterranean area. Vineyard pruning biomass has been shown to be the most difficult to properly pelletize, and all the trials reveal important drawbacks in terms of mechanical durability and bulk density [85,86]. Pellets produced from hazelnut and olive pruning showed problems related to high ash content, even if satisfactory heating values were achieved [87,88,89]. Excessive ash content, as well as high levels of chlorine and nitrogen, was revealed for olive pruning pellets produced in Greece, which, however, achieved class B for domestic use overall [81].

Pellet produced from apple pruning likewise showed excessive ash content (2.2%), achieving only I3 standards of quality for industrial applications, and the authors reported the possibility of improving the overall quality by blending apple pruning residues with coniferous sawdust [90].

The results are rather similar for tropical fruit species. Although achieving satisfactory heating values and pollutant contents for guava and avocado pruning pellets [91,92], the low lignin content did not allow satisfactory levels of bulk density and mechanical durability.

In summary, pruning has been confirmed to be a greatly challenging feedstock for pellet production at a small-scale level as well. The major problems related to high ash content mean that a small-scale supply chain for pellet production would only be efficient in the case of wide availability of low-cost (or, even better, zero-cost) feedstock concentrated in a very small area. In this way, the higher maintenance costs related to the high ash content of pruning pellets would be balanced by very low pellet production costs.

Concerning the costs of pellet production, a recent review highlighted how the costs of the feedstock do not show high variability among the different types of biomass, and, therefore, the differences in the supply chain are more related to the costs for biomass transport [93] and biomass drying [94]. Indicatively, to be competitive, the costs for pellet production should not be higher than 120 EUR/t; therefore, the theoretical supply chain for producing pellets from alternative biomass should be shaped in such a way as to not exceed this threshold.

## 6. Conclusions

Pellets are generally produced from coniferous sawdust derived from debarked stems. This feedstock ensures the best possible quality considering the high lignin content, which has strong binding and high heating values. However, in the effort to develop small-scale pellet production useful to sustain the economy and energy independence of rural areas, it is important to find alternative feedstocks. These should be able to be used to produce pellets without additives and without biomass pre-processing, making it possible to keep the cost for the feedstock at a low level. There has been interest in evaluating the properties of pellets produced from alternative forest biomass; namely, different kinds of residues and broadleaf species and pruning biomass. It is obvious that these raw materials would not make it possible to achieve the same quality standards as coniferous sawdust pellets, but this review aimed to summarize the current and recent knowledge on the topic, trying to indicate what could be the most interesting feedstocks for the development of small-scale pellet production.

The obtained results indicated that the most common concerns when producing pellets from alternative feedstocks are low bulk density and mechanical durability. These drawbacks were found for all the investigated typologies of alternative biomass and also emerged as a consequence of the fact that, in small-scale production, low-performance pelletizers are used, which do not allow the achievement of satisfactory mechanical properties. Pellets produced from pruning, coniferous residual material, and wood from short-rotation coppice plantations revealed important drawbacks regarding ash content, as well as, in some cases, the presence of nitrogen, sulfur, and chlorine.

Considering the above, the findings from the literature suggest wooden material from broadleaf species, like beech and oak, retrieved during the usual forest management activities as the best possible feedstock for small-scale pellets production. These feedstocks are able to produce pellets with low ash content, even if they have low mechanical properties. Given that the utilization of pellets with high ash content is a major drawback in small-scale production and it can strongly increase the maintenance costs, the cost-effectiveness of the overall supply chain could be compromised. On the other hand, pellets with low bulk density and mechanical durability can be successfully applied for a small-scale supply chain on the condition that the distance for transport is not great and the storage times are limited.

Finally, as future research suggestions, we suggest that more studies address the evaluation of the overall sustainability of the pellet value chain. It would be useful and appropriate to include in the pellet qualification some indicators or indices related to the sustainability of production, as these factors are more and more likely to influence the market, as well as the choice of the individual consumer.

## Figures and Tables

**Table 1:** Some of the requirements according to the standard ISO 17225-2.

Parameter	Commercial and Residential Use			Industrial Use		
	A1	A2	B	I1	I2	I3
Moisture (%)	<10	<10	<10	<10	<10	<10
Ash (%)	≤0.7	≤1.2	≤2	≤1	≤1.5	≤3
Mechanical durability (%)	≥97.5	≥97.5	≥96.5	≥97.5	≥97.5	≥96.5
Fines (%)	≤1	≤1	≤1	≤4	≤5	≤6
Additives (%)	≤2	≤2	≤2	≤3	≤3	≤3
Lower heating value (LHV—MJ/kg)	≥16.5	≥16.5	≥16.5	≥16.5	≥16.5	≥16.5
Bulk density (kg/m^2^)	≥600	≥600	≥600	≥600	≥600	≥600
Nitrogen (%)	≤0.3	≤0.5	≤1	≤0.3	≤0.3	≤0.6
Sulfur (%)	≤0.04	≤0.05	≤0.05	≤0.05	≤0.05	≤0.05
Chlorine (%)	≤0.02	≤0.02	≤0.03	≤0.03	≤0.05	≤0.1
Arsenic (mg/kg)	≤1	≤1	≤1	≤2	≤2	≤2
Cadmium (mg/kg)	≤0.05	≤0.05	≤0.05	≤1	≤1	≤1
Chromium (mg/kg)	≤10	≤10	≤10	≤15	≤15	≤15
Copper (mg/kg)	≤10	≤10	≤10	≤20	≤20	≤20
Plumb (mg/kg)	≤10	≤10	≤10	≤20	≤20	≤20
Mercury (mg/kg)	≤0.1	≤0.1	≤0.1	≤0.1	≤0.1	≤0.1
Nickel (mg/kg)	≤10	≤10	≤10	-	-	-
Zinc (mg/kg)	≤100	≤100	≤100	≤200	≤200	≤200

## Data Availability

This was a systematic literature review that did not have any data behind its development.
